# Estimate of Leaf Area Index in an Old-Growth Mixed Broadleaved-Korean Pine Forest in Northeastern China

**DOI:** 10.1371/journal.pone.0032155

**Published:** 2012-03-09

**Authors:** Zhili Liu, Guangze Jin, Yujiao Qi

**Affiliations:** Center for Ecological Research, Northeast Forestry University, Harbin, China; DOE Pacific Northwest National Laboratory, United States of America

## Abstract

Leaf area index (LAI) is an important variable in the study of forest ecosystem processes, but very few studies are designed to monitor LAI and the seasonal variability in a mixed forest using non-destructive sampling. In this study, first, true LAI from May 1^st^ and November 15^th^ was estimated by making several calibrations to LAI as measured from the WinSCANOPY 2006 Plant Canopy Analyzer. These calibrations include a foliage element (shoot, that is considered to be a collection of needles) clumping index measured directly from the optical instrument, TRAC (Tracing Radiation and Architecture of Canopies); a needle-to-shoot area ratio obtained from shoot samples; and a woody-to-total area ratio. Second, by periodically combining true LAI (May 1^st^) with the seasonality of LAI for deciduous and coniferous species throughout the leaf-expansion season (from May to August), we estimated LAI of each investigation period in the leaf-expansion season. Third, by combining true LAI (November 15^th^) with litter trap data (both deciduous and coniferous species), we estimated LAI of each investigation period during the leaf-fall season (from September to mid-November). Finally, LAI for the entire canopy then was derived from the initial leaf expansion to the leaf fall. The results showed that LAI reached its peak with a value of 6.53 m^2^ m^−2^ (a corresponding value of 3.83 m^2^ m^−2^ from optical instrument) in early August, and the mean LAI was 4.97 m^2^ m^−2^ from May to November using the proposed method. The optical instrument method underestimated LAI by an average of 41.64% (SD = 6.54) throughout the whole study period compared to that estimated by the proposed method. The result of the present work implied that our method would be suitable for measuring LAI, for detecting the seasonality of LAI in a mixed forest, and for measuring LAI seasonality for each species.

## Introduction

Leaf area index (LAI) is one of the most important characteristics of plant canopy structure and has attracted many scholars' attention [1]. LAI is defined as half the total green leaf area per unit ground surface area (m^2^ m^−2^) [2], and it directly influences both the amount of solar radiation that can be intercepted and the plant-atmosphere exchange of CO_2_, O_2_, water and energy [3]–[5]. LAI is required as an input variable in most ecosystem models simulating carbon and water cycles [6], and it often serves as a convenient surrogate measure of gross primary productivity (GPP) [7]. In addition, an accurate measurement of LAI is essential for converting leaf-level processes to the canopy level [8].

Traditional measurements of LAI are generally divided into direct and indirect methods [9]–[12]. Direct methods include destructive sampling, allometry, and litter traps [13]. Destructive sampling method is the best method to obtain LAI, but it is not suitable for measuring LAI in a large area and for dynamic monitoring because it is destructive and time-consuming, and we cannot repeatedly destroy sample forest stands [14]. An allometric approach can replace destructive sampling, but it remains difficult to monitor seasonal changes [15], [16]. Litter traps method is non-destructive, and collecting leaf litter to determine LAI is very accurate [17], [18]. However, litter traps method is more successful in deciduous forests that have a single leaf-fall season than in evergreen or mixed forests that have more continuous leaf loss and replacement, and it provides little information about LAI during the leaf-expansion season. As a result, litter traps method should not be used to monitor LAI seasonality in a mixed forest stand [15], [19].

The indirect ground method (optical instruments) infers LAI by measuring radiation transmission through the canopy [20]. The main instruments include Tracing Radiation and Architecture of Canopies (TRAC), LAI-2000 Plant Canopy Analyzer, Sunfleck ceptometer, DEMON and Hemispherical photography [10], [21]. For a mixed forest, it is possible to overcome some problems with direct methods by using this method. However, LAI is calculated by most optical instruments with the assumption that leaves have a random spatial distribution, and it is difficult to distinguish foliage from woody tissue. Thus, we use the term “effective LAI (L_e_)” to describe LAI derived optically [22]. To find true LAI, L_e_ from optical instruments must be calibrated properly (including woody tissue and the clumping effect).

In this study, we propose a practical field measurement method for LAI in the canopy of a mixed forest using non-destructive sampling. We implemented this method to obtain LAI for the entire canopy from the initial leaf expansion to the leaf fall in an old-growth mixed broadleaved-Korean pine forest in northeast China. Based on the results from the proposed method, the accuracy of the conventional indirect optical method (Hemispherical photography) for measuring LAI was investigated.

## Materials and Methods

### Ethics Statement

The field studies was conducted at the Liangshui National Nature Reserve (47°10′50″N, 128°53′20″E), which is located on the south side of the Xiaoxing'an Mountains, in northeastern China. It is a practice base for the researchers (including students and teachers) of the Northeast Forestry University. Thus we could conduct experiments there without specific permits. The experiments conducted in this study do not involve or impact endangered or protected species.

### 2.1 Study site

The study site is an old-growth, mixed broadleaved-Korean pine forest in the Liangshui National Nature Reserve, which was established in 1980, and joined China's Man and the Biosphere Reserve Network in September 1997. It was promoted to a national nature reserve with the approval of the Chinese State Council in December 1997 to protect the old growth mixed broadleaved-Korean pine forest ecosystem. The topography is complex, and the highest mountain elevation is 707.3 m. The annual mean air temperature and the mean annual rainfall are −0.3°C and 676 mm, respectively. The site is covered by snow for 130–150 days, and the frost-free period is 100–120 days. The zonal vegetation is mixed broadleaved-Korean pine forest. The species composition of the tree canopy at the study site are as follows: the needle tree group (evergreen species) consists of *Pinus koraiensis*, *Abies nephrolepis*, and *Picea* spp., with a mean diameter at breast height (DBH) of 27.58 cm and a density of 313 trees ha^−1^; the broad-leaved species (deciduous species) mainly include *Acer mono*, *Betula costata*, *Fraxinus mandshurica*, *Tilia amurensis*, *Ulmus laciniata*, *Acer tegmentosum*, *Acer ukurunduense*, *Ulmus japonica*, *Tilia mandshurica*, *Corylus mandshurica*, and *Prunus padus*, with a mean DBH of 8.36 cm and a density of 1391 trees ha^−1^.

The study was conducted in a permanent sampling plot of an area of 9 ha (300 m×300 m), divided into 900 sub-plots, 10 m×10 m each. We measured DBH and tree height and recorded the coordinates of all plants with DBH≥2 cm in each sub-plot. Aluminum tree brands with tree numbers were nailed 1.4 m above the root; however, brands were fixed by copper wire for plants with DBH<8 cm to reduce the influence on plant growth [23]. At the center (160 m×160 m) of the permanent sampling plot, litter traps and hemispherical photography were performed at the same points, on a 8×8 grid (64 total sample points) with each point separated by 20 m.

LAI observations were carried out from early May to November 15, 2007. For details, the observations for collecting litter and taking hemispherical photographs were all made on May 1^st^, May 14^th^, May 22^nd^, May 30^th^, June 5^th^, June14^th^, July 1^st^, July 15^th^, August 1^st^, September 1^st^, September 15^th^, October 1^st^, October 15^th^, November 1^st^ and November 15^th^. The leaf seasonality observations were made from May to July (contemporaneous with collecting the litter or taking hemispherical photographs).

### 2.2 Optical leaf area index

In our study, hemispherical photographs of the sample points were taken 1.3 m above ground using a WinSCANOPY 2006 Plant Canopy Analyzer (contains a digital camera (Coolpix 4500, Nikon, Tokyo, Japan), a 180° fisheye lens (Nikon FC-E8), self-leveling gimbals and a tripod). The photographs were taken with automatic exposure under uniform sky conditions, such as shortly before sunset or sunrise or when it was evenly overcast. We estimated L_e_ using hemispherical photography from May 1^st^ to November 15^th^, which has been widely utilized to measure canopy structures [24]–[26], using the software DHP (Digital Hemispherical Photography) [27]. To avoid missing small gaps in DHP at large zenith angles, L_e_ was calculated using a zenith angle range of 0–60°, although the hemispherical photographs cover the range of 0–90°.

### 2.3 The proposed method

Our method combines three components. First, L_e_ of May 1^st^ and November 15^th^ are calibrated using the three components (the woody-to-total area ratio, α; the element clumping index, Ω_E_; the needle-to-shoot area ratio, *γ_E_*), and then they represent true LAI of May 1^st^ and November 15^th^, respectively. Second, by periodically combining true LAI (May 1^st^) with the seasonality of LAI for deciduous and coniferous species throughout the leaf-expansion season (from May to August), we can estimate LAI of each investigation period in the leaf-expansion season. Third, by combining true LAI (November 15^th^) with litter trap data (both deciduous and coniferous species), we can estimate LAI of each investigation period during the leaf-fall season (from September to mid-November). LAI for the entire canopy then can be derived accordingly from the initial leaf expansion to the leaf fall.

#### 2.3.1 From L_e_ to true LAI for May 1^st^ and November 15^th^

Most optical instruments measuring LAI assume a spatially-random distribution of foliage elements [22]. In this case, L_e_ can be calculated from the gap fraction by adopting Miller's theorem [28], summarized in the following equation:
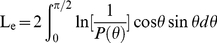
(1)where *P*(*θ*) is the gap fraction at the zenith view angle. However, most leaves in plant canopies are not randomly distributed in space. Their distribution is in close relation to the distribution of tree crowns and branches in forests [29], especially for conifers, the clumping effect beyond and within the shoots and woody tissue must be taken into account. Based on the development and validation of this theory [22], [29], true LAI (L) is calculated using the following equation:
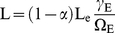
(2)where α is the woody-to-total area ratio, *γ_E_* is the needle-to-shoot area ratio quantifying the effect of foliage clumping within the shoots, Ω_E_ is the element clumping index quantifying the effect of foliage clumping at scales larger than the shoots, and L_e_ is effective leaf area index that directly obtained from optical instruments. Thus we could obtain true LAI of May 1^st^ and November 15^th^ using equation (2), with the effects of the broadleaves ignored (because there are almost no leaves for deciduous species at those times).

The woody tissue was differentiated from the greenery using Photoshop software. The clumping effect included clumping beyond and within the shoots, and the former was quantified by a clumping index directly obtained from DHP and TRAC, whereas the latter was quantified by a needle-to-shoot area ratio obtained through laboratory analysis of shoot samples following the method described in Chen [22].

##### Measurement of woody-to-total area ratio

The woody-to-total area ratio (α) equals the woody area index (WAI) divided by the plant area index (PAI). Traditional measurements of the ratio are generally divided into direct and indirect methods [9], [15], [30]. The direct destructive sampling method usually involves measuring the woody area of representative trees within forest stands, similar to the procedure used for direct measurements of LAI. However, the direct method was impossible because it was time-consuming and labor-intensive, and particularly, logging is prohibited in national nature reserves. Indirect methods (e.g., LAI-2000 or Hemispherical photography) are convenient and efficient, taking measurements during the leafless periods, but there are no leafless periods in a mixed forest stand, thus green and non-green materials cannot be separated optically. In this study, almost no broad leaves existed on May 1^st^ and November 15^th^, so the influence on the deciduous species could be ignored. We removed the errors from woody tissue with Photoshop 7.0 software. When processing the hemispherical photographs from May 1^st^ and November 15^th^, first, we obtained the total LAI (L_total_) of photograph using DHP software; second, a generic badge tool was used to replace the stems and branches (both deciduous and evergreen) with nearby background parts, then we obtained LAI of green materials (L_green_) of photograph using DHP software with the same threshold as above; third, the woody-to-total area ratio was then derived accordingly (α = (L_total_−L_green_)/L_total_). Then, the mean values of May 1^st^ and November 15^th^ were 0.096 and 0.092, respectively.

##### Observation of clumping index

The effect of foliage clumping beyond the shoots is considered using a clumping index (Ω_E_) because most of the branches and leaves of plant canopies are not randomly distributed. With the development of technology, the method developed by Chen and Cihlar [31] (briefly CC method) has been widely used to calibrate the clumping effect beyond the shoots. The final equation based on the gap size distribution theory for calculating Ω_E_ is:
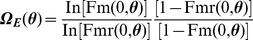
(3)where F_m_(0,*θ*) is the total canopy gap fraction at zenith angle*è*, i.e. the accumulated gap fraction from the largest to smallest gaps; and F_mr_(0,*è*) is the total canopy gap fraction after removing large gaps resulting from the non-random foliage element distribution due to canopy structures such as tree crowns and branches.

The clumping index (Ω_E_) for calibrating the clumping effect beyond the shoots can be estimated using DHP and TRACWin 3.9.1 software, and this method has been validated [15]. Then, the hemispherical photographic images from May 1^st^ and November 15^th^ were calibrated using a clumping index. The clumping index from the DHP was computed within the zenith angle range of 40–45°.

##### Measurement of needle-to-shoot area ratio

The needle-to-shoot area ratio (γ_E_) was used to quantify the clumping effect within the shoots [29]. The γ_E_ was measured according to Chen's method [22]. The clumping effect within the shoots in conifers was mostly determined by the tree's growth condition, so we tried to sample within different conditions. To obtain an average γ_E_ value for the stand, *P. koraiensis* at the study site were first grouped into three categories by their DBH: dominant (D), co-dominant (M) and suppressed (S), and three trees were selected from each class. From each tree, shoot samples (a shoot was the sampling unit) were taken at three heights: top (T), middle (M) and low (L), and nine shoots were sampled from each class, thus creating nine shoot classes: DT, DM, DL, MT, MM, ML, ST, SM and SL, for the stand. Therefore, we obtained a total of 243 shoot samples, which were taken back to the laboratory for further analysis. Based on the theoretical development by Chen [22], the needle-to-shoot area ratio (γ_E_) is calculated as follows:
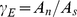
(4)where *A_n_* is half the total needle area (including all sides) in a shoot and *A_s_* is half the shoot area. *A_n_* was obtained by the volume displacement method described by Chen [22].

Chen's approach [22] was used to measure the projected shoot area (*A*_p_) at just three camera incidence angles: 0°, 45° and 90°. The following equation is used to calculate half of the total shoot area (*A*_s_):
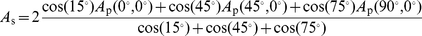
(5)


We obtained γ_E_ by combining equation (4) with (5). For *Picea* spp. and *A. nephrolepis* and deciduous forests, individual leaves were considered to be foliage elements, so γ_E_ = 1.

#### 2.3.2. Seasonality of LAI in the leaf-expansion season

##### Leaf (needle) seasonality observations

We carried out leaf (needle) seasonality observations with periodic in situ observations of sample foliage from 14 species in leaf-expansion season. Under the influence on light, water conditions and nutrients, the growth rate of single foliage may be influenced by its position within the canopy and the stand. Three trees were sampled from each species within different conditions, and five leaves for broad-leaved species and fifteen needles for coniferous species were chosen from each direction (east, south, west and north), with different heights, for one tree. On May 1^st^, May 14^th^, May 22^nd^, May 30^th^, June 5^th^, June 14^th^, July 1^st^ and July 15^th^, we obtained the size (length and width or thickness) of each sample leaf (needle), and the elements of the sample needles were measured in several places.

For broad-leaved species, the area of a single leaf is not easily multiplied length by width because of the irregular shape of the leaves. Thus we used an adjustment coefficient to adjust the leaf area based on the length and width of single leaf. To obtain the adjustment coefficient (m), the following equation is used:
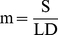
(6)where S is the area of a single leaf, L is the length of the leaf, D is the width of the leaf. To obtain the values, 20 leaves were collected from each species, and we were able to calculate the half leaf area by scanning. We obtained m value by combining the half leaf area with the length and width from observations. The mean leaf area of each species was obtained periodically by combining the length and the width from observations with m value that was assumed to be unchanged in the leaf-expansion season.

For coniferous species, the width and thickness of needles, which were determined through periodic monitoring, did not change dramatically. Therefore, we can assume that the width and thickness of needles kept the same value during the leaf-expansion season, and the area of single needle can be calculated as follows:
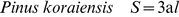
(7)

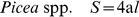
(8)where *S* is the area of single needle, a is the side of the cross-section, the values are 1.00 mm and 0.98 mm, respectively, and *l* is the length of the needle. The value of *l* is obtained from periodic observations.
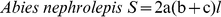
(9)where *S* is the area of a single needle, b is the width of a needle, the value is 1.33 mm, c is the thickness of the needle, the value is 0.44 mm, and *l* is the length of needle. The value of *l* was also determined from periodic observations. By periodically obtaining in suit observations of sample needles, we could estimate the mean area of a single needle of each coniferous species using equations (7)–(9).

##### Seasonality of LAI for deciduous species

For broad-leaved species, if the mean leaf area and the number of leaf for each species during the leaf-expansion season are obtained, we can estimate LAI of each species for one sample point. Assuming that the total leaf number remains the same throughout the leaf-expansion season, thus, the total leaf number can be obtained by combining little-trap data (the total leaf area of litter of the whole study period) with the mean leaf area of mature leaves (that from late leaf-expansion season) for each species that is calculated by equation (6). The total leaf area for each species could be estimated using the following two equations:
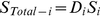
(10)

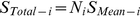
(11)where *S_Total-i_* is the total leaf area of species *i* in the late leaf-expansion season (the maximum LAI period), *D_i_* is the total mass of species *i* throughout the whole study period, *S_i_* is the SLA of species *i*, *N _i_* is the total leaf number of species *i* in the leaf-expansion season, and *S_Mean-i_* is the mean leaf area for mature leaves of species *i.* With equations (6), (10) and (11) and the litter trap area, we can then calculate LAI seasonality of all broad-leaved species on each sample point during the leaf-expansion season.

##### Seasonality of LAI for coniferous species

For coniferous species, we can similarly assume that the total needle number remains the same in the leaf-expansion season. Based on the mean leaf area of mature needle of each species for one sample point that can be estimated using equations (7)–(9), by combining the increased total LAI for each species throughout the investigation period, we can calculate the total needle number. This increased total LAI can be estimated from the needle growth and fall during investigation period for coniferous species using the following equation:
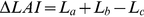
(12)where *L_a_* is true LAI of November 15^th^, *L_b_* is the total LAI from the needle litter (from May 1^st^ to November 15^th^), and *L_c_* is true LAI of May 1^st^.

The increased total LAI of each coniferous species is obtained by combining equation (12) with the ratio of LAI from the litter of each to all species throughout the investigation period. Then, we can obtain the total leaf area of each coniferous species on each sample point using the following equation:
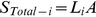
(13)where *S_Total-i_* is the total leaf area of coniferous species *i*, *L_i_* is the increased total LAI of coniferous species *i*, and *A* is the area of the litter trap. By adding equations from (7)–(13), we can estimate LAI seasonality of all coniferous species on each sample point during the leaf-expansion season.

Then, combining LAI of broad-leaved species with conifers, we can estimate the increased LAI of the whole canopy from the start of the investigation period through using the following equation:
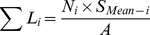
(14)where *L_i_* is the increased LAI of species *i*, *N_i_* is the total leaf (needle) number of species *i*, *S_Mean-i_* is the mean leaf (needle) area of species *i*, and *A* is the total area of all sampling areas (litter traps).

For example, when true LAI of May 1^st^ as a benchmark is added to the increased LAI of May 14^th^, minus LAI from the needle litter during the period, we obtain true LAI of May 14^th^. By analogy, true LAI of the study stand can be obtained for other sampling times during the leaf-expansion season.

#### 2.3.3 Seasonality of LAI in the leaf-fall season

##### Observation of specific leaf area

Specific leaf area (SLA) (the leaf area per unit of dry mass, cm^2^ g^−1^) is determined in relation to the species and living conditions of sample trees, and to the positions of sample leaves within the crown [32]–[34]. However, SLA (single species) has been shown to change only slightly during the leaf-expansion season [35]. To accurately obtain SLA of each species, the above factors were considered when sampling the mature leaves (needles) of the main species.

Non-flat leaves were not collected when sampling broad leaves. The flat one-sided areas of leaves were measured by scanning. The area was calculated by counting the number of leafy pixels and multiplying the number by the pixel size. To reduce error, SLA of uncertain broad-leaved fragments from the litter was obtained from the mean SLA of other broad-leaved species.

Needle age (current year versus one-year-old needles) was considered when sampling needles [36], [37]. The areas of needles were measured by the volume displacement method [22]. First, the whole shoot with the stem was immersed in water in a container that was resting on a sensitive balance and that was large enough to prevent the shoot from touching the side or the bottom of the container (moderate detergent was put into the water to reduce the water's surface tension). The displaced water volume is measured as the increase in weight if the shoot is not touching the side or the bottom of the container because the displaced water exerts forces equally in all directions including the bottom of the container. Then, we could obtain the entire shoot volume (*V_1_*) through increasing the weight. Second, the stem volume (*V_2_*) was measured in the same way, with the needles removed, and simultaneously, the number of needles and the average length of the needles were measured. To obtain the volume of the needles (*V*), the total volume was reduced by the stem volume, briefly 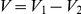.

The top of the needles was found to be acuminate, and we could ignore the areas at the top because they were negligibly small. The shape of *P. koraiensis* approximates a triangular prism, and *Picea* spp. and *A. nephrolepis* are cuboid. The equations of half the total needle area are easily obtained for *P. koraiensis* and *Picea* spp. because their cross-sections are an equilateral triangle and square, respectively. For *A. nephrolepis*, the ratio width and thickness of each needle were measured by Vernier calipers. To reduce the error, the width and thickness were taken as the average of 1/4, 1/2 and 3/4 of each needle. The equations of half the total needle area (*A*) are calculated as follows:
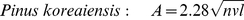
(15)

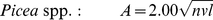
(16)

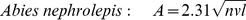
(17)where *v* is the displaced volume (cm^3^ or g) of the needles in a shoot, *n* is the total number of needles submerged, and *l* is the average length (cm).

The mass of all the sampling leaves (needles) was measured after drying (65°C for 48 h). Then, we can calculate SLA using the following equation:
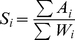
(18)where *S_i_* is the specific leaf area of species *i*, *A_i_* is the area of species *i*, and *W_i_* is the dry mass of species *i.*

##### Litter trap observations

Each litter trap had a square aperture of 0.5 m^2^, and its base was approximately 0.5 m above the ground. From May to September, litter was recovered at the same time that the leaf expansion was surveyed, and from September to mid-November, litter was recovered bimonthly. During each litter collection, we sorted the litter in each trap into the leaves of each species in time to avoid affecting the results due to the decomposition of the leaves. After the litter was weighed, the sampled leaves were dried at 65°C for 48 h, and the total dry mass of all the leaves was obtained. LAI was then calculated through SLA. The leaves of the deciduous species from May to August could be discarded because the number of leaves collected was small. In fact, LAI from deciduous species during that period occupied only 0.04% of the leaves collected during the whole study period.

Therefore, when true LAI of November 15^th^ is used as a benchmark and is added to LAI of November 15^th^ from the litter (both deciduous and coniferous species), LAI of November 1^st^ is obtained. By analogy, true LAI of the study stand can be obtained for other sampling times during the leaf-fall season.

Finally, by adding the component LAI of all species, we can then obtain true LAI for the entire canopy from the initial leaf expansion to the leaf fall.

## Results

### 3.1 Clumping index

The clumping index of each sample point on May 1^st^ and November 15^th^ was directly obtained from DHP and TRACWin 3.9.1 software, and the difference in the results was not significant (Table 1).

**Table 1:** Element clumping index, quantifying the effect of foliage clumping at scales larger than the shoot, as a function of the solar zenith angle for the study stand.

Date	Mean ± SD	Maximum	Minimum	Sample
May 1^st^	0.91±0.04	1.00	0.80	64
November 15^th^	0.91±0.04	0.97	0.81	64

Note: All the values of clumping index were unitless.

### 3.2 Needle-to-shoot area ratio (γ_E_)

The values of γ_E_ for the *P. koraiensis* ranged from 1.48 to 2.68, and the standard deviation of each γ_E_ was less than 0.52 (Table 2). The average γ_E_ increased with the increasing height level, but the variations found within the top were dominant, co-dominant and suppressed, and the values were 2.37 (SD = 0.48), 1.92 (SD = 0.40) and 1.81 (SD = 0.37), respectively. However, the variations within the middle and low portion of each canopy class were small, and the mean value was 1.64 (SD = 0.04). Generally, dominant trees had the largest values, followed by co-dominant and suppressed trees. These large differences were mostly determined by the growth conditions, such as light and water [32], suggesting that the separation of the canopy classes was necessary for the sampling strategy. In this study, the mean value of 1.77 was used as the needle-to-shoot area ratio of the *P. koraiensis.*

**Table 2:** Mean needle-to-shoot area ratio and standard deviation of *P. koraiensis* for nine trees and height classes in north-eastern China.

Canopy	Sample	Top (T)	Middle (M)	Low (L)
Dominant (D)	a	2.68±0.33	1.78±0.15	1.56±0.15
	b	2.38±0.35	1.63±0.22	1.58±0.14
	c	2.03±0.52	1.65±0.20	1.78±0.20
Co-dominant (M)	d	1.59±0.15	1.57±0.24	1.54±0.21
	e	1.95±0.35	1.63±0.21	1.60±0.23
	f	2.23±0.37	1.85±0.22	1.64±0.25
Suppressed (S)	g	1.77±0.25	1.68±0.25	1.70±0.10
	h	2.15±0.35	1.60±0.14	1.69±0.16
	i	1.49±0.14	1.49±0.15	1.48±0.11
Mean		1.77±0.37		

Note: In the stand, 243 shoot samples were taken from nine trees: three dominant (D), three co-dominant (M), and three suppressed (S), at three heights: top (T), middle (M), and bottom (L), forming nine classes with 27 shoot samples each: DT, DM, DL, MT, MM, ML, ST, SM, and SL; all these values were unitless.

### 3.3 Specific leaf area (SLA)

SLA largely varied with tree species (Table 3). SLA of *F. mandshurica* was largest with a value of 385.96 cm^2^ g^−1^, but SLA of *Picea* spp. was only 49.84 cm^2^ g^−1^. Generally, SLA of coniferous species was smaller with a mean value of 66.60 cm^2^ g^−1^ (SD = 15.06), which was a quarter of the broad-leaved species.

**Table 3:** Specific leaf area of main tree species, obtained from sample foliage in the study stand.

Species	SLA (cm^2^ g^−1^)
*Pinus koraiensis*	79.00
*Picea* spp.	49.84
*Abies nephrolepis*	70.96
*Acer mono*	315.16
*Fraxinus mandshurica*	385.96
*Tilia amurensis*	163.3
*Betula costata*	197.44
*Acer tegmentosum*	241.28
*Corylus mandshurica*	382.94
*Acer ukurunduense*	378.99
*Ulmus laciniata*	300.16
*Tilia mandshurica*	354.49
*Ulmus japonica*	212.42
*Prunus padus*	123.95
*Populus ussuriensis*	125.78
*Quercus mongolica*	280.05

Note: Specific leaf area (SLA) of uncertain broad-leaved fragments from the litter was obtained from the average SLA of other broad-leaved species.

### 3.4 Adjustment coefficient

Slight differences were found within species because of the different shapes (Table 4). The adjustment coefficient values for the study species ranged from 0.48 to 0.74, and an average value was 0.65 (SD = 0.08). We found that the shape of the leaves determined the value of adjustment coefficient, the product of length and width of heart-shaped (or elliptical) leaves was more close to true leaf area (namely the adjustment coefficient value was bigger) than leaves of other shapes, such as in *T. amurensis* and *T. mandshurica*. However, the palm-shaped *A. mono* had an adjustment coefficient value of only 0.48.

**Table 4:** Adjustment coefficient, adjusting the leaf area based on the length and width of single leaf, obtained from 20 sample leaves for each broad-leaved species in the study stand.

Species	Adjustment	SD
	coefficient	
*Prunus padus*	0.72	0.05
*Betula costata*	0.62	0.01
*Acer ukurunduense*	0.54	0.09
*Tilia mandshurica*	0.73	0.02
*Ulmus laciniata*	0.64	0.04
*Corylus mandshurica*	0.72	0.03
*Acer tegmentosum*	0.67	0.04
*Fraxinus mandshurica*	0.63	0.02
*Acer mono*	0.48	0.03
*Ulmus japonica*	0.62	0.04
*Tilia amurensis*	0.74	0.06

Note: All the values of adjustment coefficient were unitless.

### 3.5 Leaf seasonality observations

**Figure 1:**
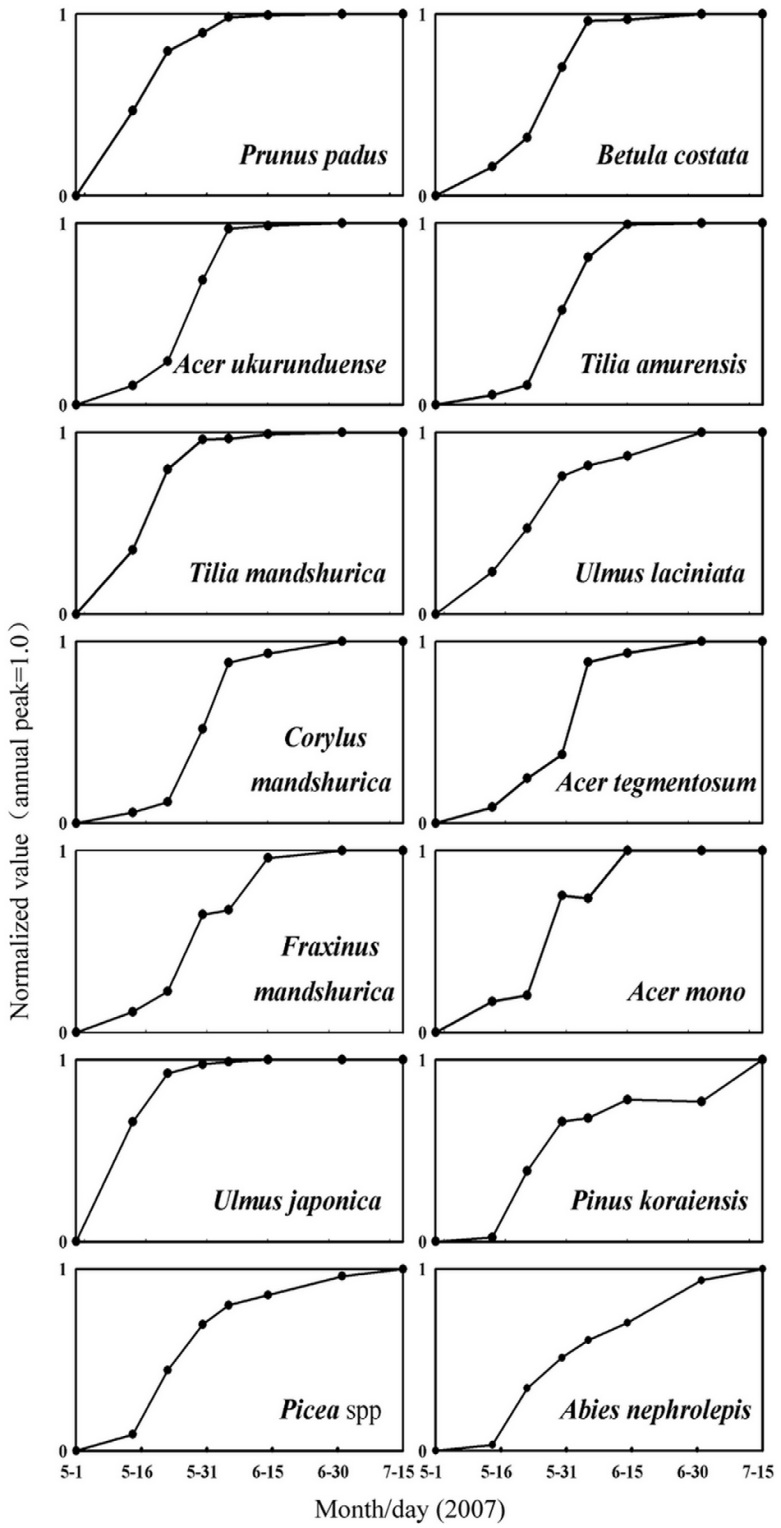
Seasonality of mean leaf area for the tree species, obtained from 60 sample leaves (needle samplings were moderately increased) of 3 individuals for each species. Each time series for the data was normalized using the annual maximum value set to 1.0.

All the species showed clear seasonality of the single leaf area (Figure 1). As seen in the changing leaf area of the species, most species except *F. mandshurica* and *A. mono* had a single leaf flush. The first species that reached leaf flush were *U*. *japonica* and *P. padus*, lasting from early May to approximately May 22^nd^. *T. mandshurica* and *U. laciniata* reached the flush in mid-May, and it lasted approximately half a month. As of May 22^nd^, more species were reaching flush, such as *C. mandshurica*, *B. costata*, *A. ukurunduense*, *A. tegmentosum*, and *T. amurensi*s. In contrast, *F. mandshurica* and *A. mono* showed two leaf flushes: the first one around May 22^nd^ and the second in early June, each lasting for about ten days. Generally, coniferous species had a later emergence of leaves, such as *P. koraiensis* and *Picea* spp., and were all after mid-May. Only the growth of *A. mono* was irregular (the leaf area decreased in late May and recovered in mid-June), probably because of herbivory by insects (mainly aphids), and when the leaves in the second flush grew larger than those eaten by insects, the single leaf area recovered.

### 3.6 Litter trap observations

The total area from litter of all species collected throughout the investigation period was 1.65 m^2^, and the area from the main species showed clear differences (Table 5). Generally, the area from the broad-leaved species was about three times as large as that of the coniferous species. However, *P. koraiensis* accounted for 21.35% (maximum ratio) of the total area. The standard error of the total area per species were all less than 0.04, suggesting that the experimental strategy (the number of traps (64) and measurement of SLA) was reliable for the study site.

**Table 5.Total:** area from litter accounted for by the major species at the study site in the whole investigation period.

Species	Area (m^2^) ± SE	Fraction (%)
*Prunus padus*	0.00±0.00	0.11
*Ulmus japonica*	0.02±0.01	1.49
*Abies nephrolepis*	0.03±0.00	1.70
*Acer tegmentosum*	0.03±0.01	1.83
*Picea* spp.	0.04±0.00	2.26
*Tilia mandshurica*	0.04±0.01	2.50
*Corylus mandshurica*	0.08±0.02	4.76
*Acer ukurunduense*	0.08±0.02	4.93
*Ulmus laciniata*	0.13±0.02	7.97
*Betula costata*	0.13±0.02	8.17
*Tilia amurensis*	0.16±0.03	9.84
*Fraxinus mandshurica*	0.17±0.04	10.35
*Acer mono*	0.21±0.02	13.00
*Pinus koraiensis*	0.30±0.03	21.35
other	0.16	9.70
total	1.65	100.00

Note: Number of litter traps per species n = 64.

### 3.7 LAI of main species in all seasons

**Figure 2:**
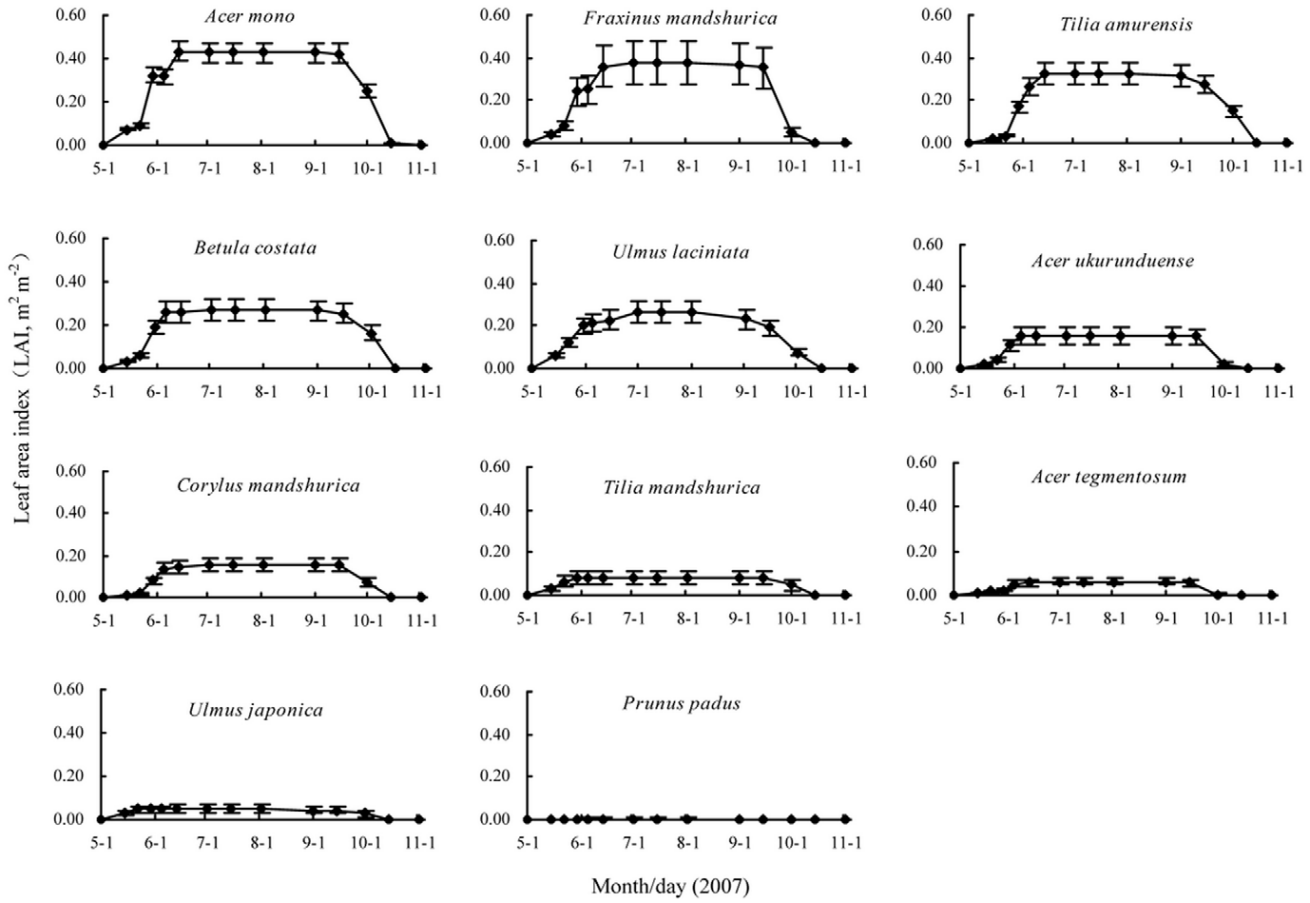
LAI of total broad-leaved species in the study site estimated by three components: true LAI of May 1^st^ and November 15^th^, litter trap data and leaf seasonality observations. Error bars represent the standard error.

By combining the three components: true LAI of May 1^st^ and November 15^th^, litter trap data and the seasonality of the leaf area, we were able to estimate LAI of the major broad-leaved species during all seasons (Figure 2). Every broad-leaved species reached its peak in July, and *A. mono* had the largest peak LAI with a value of 0.43 (SE = 0.05) m^2^ m^−2^, followed by *F. mandshurica* with a value of 0.37 (SE = 0.10) m^2^ m^−2^. *P. padu* was the smallest, only as large as approximately one percent of the largest. All broad-leaved species, except for *U. laciniata*, began to fall in early September, and *U. laciniata* fell in late August. All species had a rapid falling of leaves in late September, and the leaves of *P. padu* and *A. tegmentosum* fell in early October, but the others fell in mid-October.

**Figure 3:**
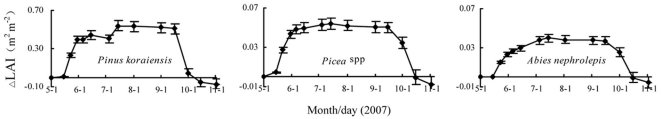
LAI of total coniferous species in the study site estimated by the increased LAI from leaf seasonality observations minus the decreased LAI from litter trap data. Error bars represent the standard error.

LAI of the three coniferous species were always greater than 0 because they were evergreen. By combining leaf seasonality observations and litter trap data, we were able to estimate the dynamic variations of LAI in increased and decreased (ΔLAI) during all seasons (Figure 3). For *Picea* spp. and *A. nephrolepis*, the changes in ΔLAI had a single flush in mid-July. However, for *P. koraiensis*, the ΔLAI decreased in mid-June and recovered in early July showed that the range of LAI increased from leaf seasonality observations was less than the range of LAI decreased from litter trap data during this period. In general, for the three coniferous species, the increased LAI from leaf seasonality observations were nearly the same as the decreased LAI from the litter trap data.

**Figure 4:**
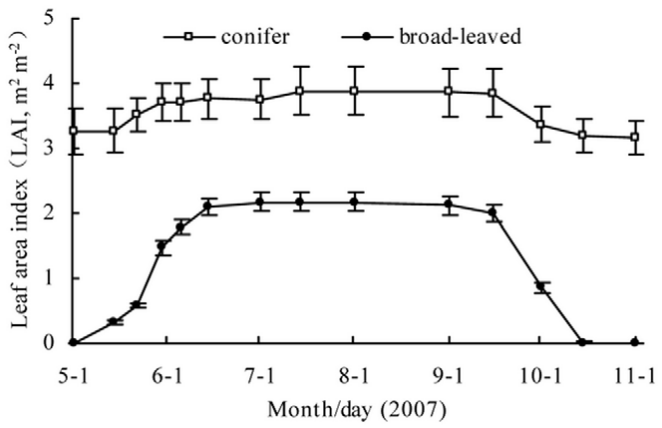
The total LAI of broad-leaved and coniferous species estimated throughout the study period. For broad-leaved species, LAI was estimated using the leaf seasonality observations and litter trap data during the study period; and for coniferous species, in addition to these data, LAI was also estimated based on true LAI on May 1^st^ and November 15^th^ because there are no leafless periods in a mixed forest. Error bars represent the standard error.

The total LAI of all broad-leaved species showed clear seasonal changes with a maximum of 2.17 m^2^ m^−2^ on July 15^th^ (Figure 4). Although the total LAI of all coniferous species had the largest peak with the value of 3.89 m^2^ m^−2^ on July 15^th^, it only increased 23% more than the minimum.

### 3.8 LAI estimation using the indirect optical method

**Figure 5:**
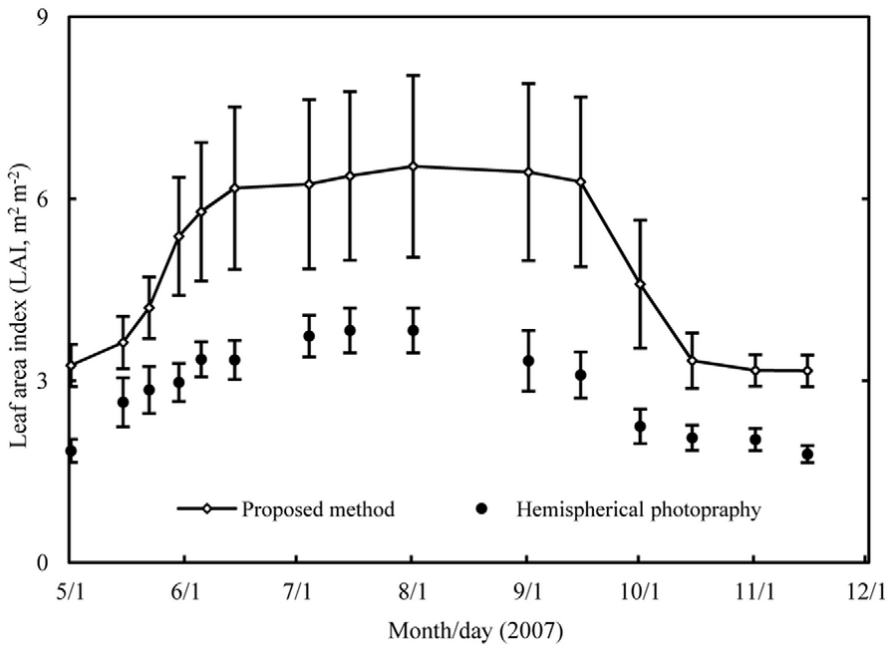
LAI of the canopy estimated by two methods: The method proposed in the present study (which was estimated by three components: LAI of May 1^st^ and November 15^th^, litter trap data and leaf seasonality observations) and the hemispherical photography. Error bars represent the standard error.

LAI estimated from the optical method for the studied species ranged from 1.79 to 3.83 m^2^ m^−2^, obviously lower (the mean underestimated 41.64%) than those provided by our method throughout the study period (Figure 5). LAI from both methods peaked in early August. Moreover, the pattern of seasonal change was different. In late May, the optical method showed a lower increasing speed than our method. From September 15^th^ to October 15^th^, the optical method showed a lower decreasing speed than our method, whereas the seasonality of LAI from the optical method showed little variation over the entire study period.

## Discussion

### 4.1 Reliability of the proposed method

In our study, 11 broad-leaved species that we selected for leaf seasonality observations accounted for 87.0% of LAI (August 1^st^) estimated from the litter trap data. This percentage suggests that the seasonality of about 13% of the total LAI was uncertain, probably a result of other broad-leaved species without leaf-expansion observations (the basal area of other broad-leaved species accounts for 12.8% of the total broad-leaved species). A similar result was shown by Nasahara et al. [38], in which species selected for the leaf seasonality observations accounted for 84% of LAI estimated from the litter trap data. Our result did not contain coniferous species because those leaves were falling during the entire study period. Therefore, we could probably measure more accurate assessments of the seasonality of LAI by obtaining leaf-expansion observations for more species in future studies.

In present study, we could not calculate the increased LAI for each coniferous species if we only used litter traps during the leaf-fall season because needles fell during the entire study period. We solved this problem successfully. First, we estimated the total LAI of all conifers from the litter collected throughout the study period, added true LAI of November 15^th^, and subtracted true LAI of May 1^st^ before obtaining the increased LAI of all coniferous species, simplified as equation (12). By combining the ratio of litter from each coniferous species of all species in the entire study period, we obtained the increased LAI of each coniferous species.

There are some weaknesses in the present study. First, we calibrated the *Picea* spp. and *A. nephrolepis* by needle-to-shoot area ratio by mistake while obtaining true LAI of May 1^st^ and November 15^th^ because the two species did not exhibit the clumping effect within the shoots. However, we determined the error, with a value of 7%, according to the litter proportion of the two species (LAI from the litter of the two species was 15.6% of the total coniferous species), and obviously, we could discard the error relative to the 41.64% underestimated by the optical method. Second, although we considered factors that could affect leaf measurements (e.g., shape, size, and growth conditions) of the sample leaves during our measurements to adjust the coefficients of all species, the sample leaves were not collected at the same time as the leaf-expansion observation; thus, measuring whether or not the adjustment coefficients were influenced by the leaf collection method was not validated in this study. Finally, we ignored the influence on perennial needles when estimating LAI. If we could eliminate these parts of the process, our method would become more accurately.

### 4.2 Measurement of main parameters

LAI obtained during the leafless period by the optical methods was assumed to represent the woody area index, which was used in previous studies. However, that is not suitable for a coniferous forest because there are no leafless periods in an evergreen forest stand [27], [30], [39]. We can measure the woody-to-total area ratio using Photoshop software and the optical method, and to overcome the huge investment in labor and time, this method is more reasonable because it is forbidden to destroy the national nature reserve. The mean values of May 1^st^ and November 15^th^ were 0.096 and 0.092, respectively. Those values conform to the published values of a range from 0.03 to 0.40 [30], [40].

The clumping effect exists not only beyond the shoots but also within the shoots (especially in conifer forest). The measurement of the clumping index (beyond the shoots) is an exciting topic, and the methods mainly include CI_LX_ (clumping index from logarithmic gap averaging); CI_W_ (clumping index from modified logarithmic gap averaging); CI_CC_ (clumping index from gap size distribution); CI_CLX_ (clumping index from combination of gap size and logarithmic averaging); and CI_PCS_ (clumping index from Pielou's coefficient of spatial segregation) [41]. We obtained the clumping index directly from the DHP-TRAC software that was widely used by other studies [39], [40]. By making comparative studies of these methods in future studies, we will determine which one is more suitable for this research site. The clumping effect within the shoots exists in conifer species and varies with the living conditions of trees [39]. To reduce the error, we took the average of a large quantity of shoot sample within different conditions. The needle-to-shoot area ratio was 1.77. Similar results have been published. For instance, Chen et al. [29] realized a needle-to-shoot area ratio that ranged from 1.4 to 1.8 for a boreal conifer forest, and Bréda et al. [15] estimated γ_E_ values of 1.2–2.0 for a stand of coniferous forest.

### 4.3 Comparison with optical method

At present, optical methods are widely used to estimate LAI and its dynamic changes because they are easier and quicker to carry out. However, the hemispherical photography technique tends to underestimate LAI [25], [42], [43]. In comparison with our method, the hemispherical photography method provided lower LAI values (a range from 27.12% to 51.07%) and a smaller seasonal variation amplitude. Underestimates of hemispherical photography have been reported in other studies. For instance, Zhang et al. [43] demonstrated that digital hemispherical photographs taken with automatic exposure are not reliable, causing *L_e_* underestimations by 16–71%, and Van Gardingen et al. [44] found that the hemispherical photography method resulted in an underestimate of 50% compared to a destructive harvest. So, it is necessary to validate and improve indirect optical methods. To learn the reasons for the discrepancy between the proposed method and optical method, we will need to validate each step in the derivation of LAI in each method and present that in a further study on this stand.

### Conclusions

This proposed method can provide not only the total LAI but also LAI for each species and its seasonal changes. By contrast, the optical method average underestimated LAI by 41.64% (SD = 6.54). Based on reasonably calibrating LAI from the optical method, by combining leaf-expansion observations with litter traps to estimate LAI and its seasonal changes in a mixed broadleaved-Korean pine forest, this method will become an effective method in the future.

## References

[1] Muraoka H, Saigusa N, Nasahara KN, Noda H, Yoshino J (2010). Effects of seasonal and interannual variations in leaf photosynthesis and canopy leaf area index on gross primary production of a cool-temperate deciduous broadleaf forest in Takayama Japan.. J Plant Res.

[2] Chen JM, Black TA (1992). Defining leaf area index for non-flat leaves.. Plant Cell Environ.

[3] Behera SK, Srivastava P, Pathre UV, Tuli R (2010). An indirect method of estimating leaf area index in *Jatropha curcas* L using LAI-2000 Plant Canopy Analyzer.. Agric For Meteorol.

[4] Sonnentag O, Chen JM, Roberts DA, Talbot J, Halligan KQ (2007). Mapping tree and shrub leaf area indices in an ombrotrophic peatland through multiple endmember spectral unmixing.. Remote Sens Environ.

[5] Sprintsin M, Karnieli A, Berliner P, Rotenberg E, Yakir D (2007). The effect of spatial resolution on the accuracy of leaf area index estimation for a forest planted in the desert transition zone.. Remote Sens Environ.

[6] Asner GP, Scurlock JMO, Hicke JA (2003). Global synthesis of leaf area index observations: implications for ecological and remote sensing studies.. Global Ecol Biogeogr.

[7] Barr AG, Black TA, Hogg EH, Kljun N, Morgenstern K (2004). Inter-annual variability in the leaf area index of a boreal aspen-hazelnut forest in relation to net ecosystem production.. Agric For Meteorol.

[8] Gower ST, Norman JM (1991). Rapid estimation of leaf area index in conifer and broad-leaf plantations.. Ecology.

[9] Deblonde G, Penner M, Royer A (1994). Measuring leaf area index with the LI-COR LAI-2000 in pine stands.. Ecology.

[10] Jonckheere I, Fleck S, Nackaerts K, Muys B, Coppin P (2004). Review of methods for in situ leaf area index determination: Part I Theories, sensors and hemispherical photography.. Agric For Meteorol.

[11] Pinto-Júnior OB, Sanches L, Lobo FdA, Brandão AA, Nogueira JdS (2010). Leaf area index of a tropical semi-deciduous forest of the southern.. Amazon Basin Int J Biometeorol.

[12] Sonnentag O, Talbot J, Chen JM, Roulet NT (2007). Using direct and indirect measurements of leaf area index to characterize the shrub canopy in an ombrotrophic peatland.. Agric For Meteorol.

[13] Ryu Y, Sonnentag O, Nilson T, Vargas R, Kobayashi H (2010). How to quantify tree leaf area index in an open savanna ecosystem: A multi-instrument and multi-model approach.. Agric For Meteorol.

[14] Macfarlane C, Grigg A, Evangelista C (2007). Estimating forest leaf area using cover and fullframe fisheye photography: thinking inside the circle.. Agric For Meteorol.

[15] Bréda NJJ (2003). Ground-based measurements of leaf area index: a review of methods, instruments and current controversies.. J Exp Bot.

[16] Marshall JD, Waring RH (1986). Comparison of methods of estimating leaf-area index in old-growth Douglas-fir.. Ecology.

[17] Neumann HH, Den Hartog G, Shaw RH (1989). Leaf area measurements based on hemispheric photographs and leaf-litter collection in a deciduous forest during autumn leaf-fall.. Agric For Meteorol.

[18] Mussche S, Samson R, Nachtergale L, De Schrijver A, Lemeur R (2001). A comparison of optical and direct methods for monitoring the seasonal dynamics of leaf area index in deciduous forests.. Silva Fennica.

[19] Cutini A, Matteucci G, Mugnozza GS (1998). Estimation of leaf area index with the Li-Cor LAI 2000 in deciduous forests.. Forest Ecol Manag.

[20] Wasseige C, Bastin D, Defourny P (2003). Seasonal variation of tropical forest LAI based on field measurements in Central African Republic.. Agric For Meteorol.

[21] Pierce LL, Running SW (1988). Rapid estimation of coniferous forest leaf area index using a portable integrating radiometer.. Ecology.

[22] Chen JM (1996). Optically-based methods for measuring seasonal variation of leaf area index in boreal conifer stands.. Agric For Meteorol.

[23] Jin GZ, Xie XC, Tian YY, Kim JH (2006). The pattern of seed rain in the broadleaved-Korean pine mixed forest of Xiao xing an Mountains, China.. J Kor For Soc.

[24] Garrigues S, Shabanov NV, Swanson K, Morisette JT, Baret F (2008). Intercomparison and sensitivity analysis of Leaf Area Index retrievals from LAI-2000, AccuPAR, and digital hemispherical photography over croplands.. Agric For Meteorol.

[25] Montes F, Pita P, Rubio A, Cañellas I (2007). Leaf area index estimation in mountain even-aged Pinus silvestris L stands from hemispherical photographs.. Agric For Meteorol.

[26] Schleppi P, Conedera M, Sedivy I, Thimonier A (2007). Correcting non-linearity and slope effects in the estimation of the leaf area index of forests from hemispherical photographs.. Agric For Meteorol.

[27] Leblanc SG, Chen JM, Fernandes R, Deering DW, Conley A (2005). Methodology comparison for canopy structure parameters extraction from digital hemispherical photography in boreal forests.. Agric For Meteorol.

[28] Miller JB (1967). A formula for average foliage density.. Aust J Bot.

[29] Chen JM, Rich PM, Gower ST, Norman JM, Plummer S (1997). Leaf area index of boreal forests: theory, techniques, and measurements.. J Geophys Res.

[30] Zou J, Yan GJ, Zhu L, Zhang WM (2009). Woody-to-total area ratio determination with a multispectral canopy imager.. Tree Physiol.

[31] Chen JM, Cihlar J (1996). Retrieving leaf area index of boreal conifer forests using Landsat TM images.. Remote Sens Environ.

[32] Bouriaud O, Soudani K, Bréda N (2003). Leaf area index from litter collection: impact of specific leaf area variability within a beech stand.. Can J For Res.

[33] Gower ST, Kucharik CJ, Norman JM (1999). Direct and indirect estimation of leaf area index, fAPAR, and net primary production of terrestrial ecosystems.. Remote Sens Environ.

[34] Nouvellon Y, Laclau JP, Epron D, Kinana A, Mabiala A (2010). Within-stand and seasonal variations of specific leaf area in a clonal Eucalyptus plantation in the Republic of Congo.. Forest Ecol Manag.

[35] Eriksson H, Eklundh L, Hall K, Lindroth A (2005). Estimating LAI in deciduous forest stands.. Agric For Meteorol.

[36] Ishii H, Ford ED, Boscolo ME, Manriquez AC, Wilson ME (2002). Variation in specific needle area of old-growth Douglas-fir in relation to needle age, within-crown position and epicormic shoot production.. Tree Physiol.

[37] Wang XC, Janssens IA, Curiel Yuste J, Ceulemans R (2006). Variation of specific leaf area and upscaling to leaf area index in mature Scots pine.. Trees-struct Funct.

[38] Nasahara KN, Muraoka H, Nagai S, Mikami H (2008). Vertical integration of leaf area index in a Japanese deciduous broad-leaved forest.. Agric For Meteorol.

[39] Chen JM, Govind A, Sonnentag O, Zhang YQ, Barr A (2006). Leaf area index measurements at Fluxnet-Canada forest sites.. Agric For Meteorol.

[40] Macfarlane C, Hoffman M, Eamus D, Kerp N, Higginson S (2007). Estimation of leaf area index in eucalypt forest using digital photography.. Agric For Meteorol.

[41] Gonsamo A, Pellikka P (2009). The computation of foliage clumping index using hemispherical photography.. Agric For Meteorol.

[42] Thimonier A, Sedivy I, Schleppi P (2010). Estimating leaf area index in different types of mature forest stands in Switzerland: a comparison of methods.. Eur J Forest Res.

[43] Zhang YQ, Chen JM, Miller JR (2005). Determining digital hemispherical photograph exposure for leaf area index estimation.. Agric For Meteorol.

[44] Van Gardingen PR, Jackson GE, Hernandez-Daumas S, Russell G, Sharp L (1999). Leaf area index estimates obtained for clumped canopies using hemispherical photography.. Agric For Meteorol.

