# Correction: Estimate of Leaf Area Index in an Old-Growth Mixed Broadleaved-Korean Pine Forest in Northeastern China

**DOI:** 10.1371/annotation/ac66c0af-51c4-4aaa-b966-2deb91cfd551

**Published:** 2012-06-26

**Authors:** Zhili Liu, Guangze Jin, Yujiao Qi

There was an error in the published funding statement. The correct funding is: This work was financially supported by the Forestry Science and Technology Supporting Program of China (No. 2011BAD37B01) (http://www.most.gov.cn/), [^] the Program for Changjiang Scholars and Innovative Research Team in University (http://www.moe.edu.cn/), [^] the National Natural Science Foundation of China (No. 30770350) (http://www.nsfc.gov.cn), [^] and by the Northeast Forestry University Graduate Thesis Funded Projects (No. GRAM09) (http://www.nefu.edu.cn). [^] The funders had no role in study design, data collection and analysis, decision to publish, or preparation of the manuscript. 

